# Using [^18^F]FDG PET/CT to Identify Optimal Responders to Neoadjuvant Therapy in Breast Cancer—Results from a Prospective Patient Cohort

**DOI:** 10.3390/cancers17132133

**Published:** 2025-06-25

**Authors:** Fabrizia Gelardi, Paola Tiberio, Rosalba Torrisi, Roberta Zanca, Marcello Rodari, Alberto Zambelli, Armando Santoro, Bethania Fernandes, Andrea Sagona, Valentina Errico, Alberto Testori, Corrado Tinterri, Arturo Chiti, Rita De Sanctis, Martina Sollini, Lidija Antunovic

**Affiliations:** 1 Department of Biomedical Sciences, Humanitas University, Pieve Emanuele, 20072 Milan, Italy; gelardi.fabrizia@hsr.it (F.G.); roberta.zanca@cancercenter.humanitas.it (R.Z.); azambelli@asst-pg23.it (A.Z.); armando.santoro@cancercenter.humanitas.it (A.S.); corrado.tinterri@hunimed.eu (C.T.); 2 Faculty of Medicine, Vita-Salute San Raffaele University, 20132 Milan, Italy; chiti.arturo@hsr.it (A.C.); sollini.martina@hsr.it (M.S.); 3 IRCCS San Raffaele Hospital, 20132 Milan, Italy; antunovic.lidija@hsr.it; 4 IRCCS Humanitas Research Hospital, 20089 Rozzano, Italy; paola.tiberio@cancercenter.humanitas.it (P.T.); rosalba.torrisi@cancercenter.humanitas.it (R.T.); marcello.rodari@cancercenter.humanitas.it (M.R.); bethania.fernandes@humanitas.it (B.F.); andrea.sagona@cancercenter.humanitas.it (A.S.); valentina.errico@cancercenter.humanitas.it (V.E.); alberto.testori@cancercenter.humanitas.it (A.T.); 5 ASST Papa Giovanni XXIII, 24127 Bergamo, Italy

**Keywords:** breast cancer, PET/CT, Neoadjuvant chemotherapy, surgery, SUVmax, TBR

## Abstract

**Background/objectives**: Pathological complete response (pCR) to neoadjuvant chemotherapy (NAC) in breast cancer (BC) is a solid indicator of favourable prognosis, potentially also being useful for more conservative patient management. We aim to explore the potential of [^18^F]FDG PET/CT as a non-invasive method to predict response to NAC. **Methods**: In this prospective, observational cohort study, we enrolled BC patient candidates for NAC who underwent baseline and preoperative [^18^F]FDG PET/CT. NAC response was determined using final histopathology. PET images were assessed qualitatively and semi-quantitatively, and the findings correlated with NAC response. **Results**: In total, 133 BC patients were included. The visual analysis of preoperative PET/CT detected residual disease (RD) with high specificity (>93%) and moderate sensitivity, based on pCR/RD classification and RCB index. Semiquantitative measures (SUVmax, TBR) were significantly higher in non-responders across the classification methods (*p* < 0.001 for all). **Conclusions**: These findings highlight the potential of preoperative [^18^F]FDG PET/CT as a complementary tool for identifying excellent responders to NAC across BC subtypes or response criteria. This could inform personalised treatment and potentially allow for surgery to be omitted in selected patients.

## Simple Summary

The emerging paradigm in breast cancer management involves potentially avoiding surgery in excellent responders to neoadjuvant chemotherapy, thereby improving quality of life, patient outcomes, and cost-effectiveness. Thus, we test, in this paper, whether baseline and/or preoperative [^18^F]Fluorodeoxyglucose ([^18^F]FDG) positron emission tomography/computed tomography (PET/CT) could identify breast cancer patients who achieve pathological complete response after neoadjuvant chemotherapy. In this prospective cohort study, the visual assessment of preoperative PET/CT had a high specificity for the identification of patients with residual disease after neoadjuvant chemotherapy. In addition, semiquantitative PET parameters in the preoperative assessment were significantly associated with response to neoadjuvant chemotherapy. Our findings demonstrate that [^18^F]FDG PET/CT has the potential to guide patient management by identifying responders who have achieved pathological complete response and those with extensive residual disease, allowing for the personalised de-escalation of surgical strategies.

## 1. Introduction

Neoadjuvant chemotherapy (NAC) is currently offered to locally advanced and unresectable breast cancer (BC) [1], and recently it has also emerged as a standard treatment option for patients with operable disease who are eligible for adjuvant chemotherapy [1,2]. The primary purposes of NAC are twofold: to reduce mortality and to expand surgical options. Additionally, NAC provides in vivo information about tumour chemosensitivity, contributes to defining the adjuvant treatment plan, and guides risk stratification by evaluating treatment efficacy through pathological response assessment [3]. Different methods have been developed to standardise the assessment of pathological response to NAC [4,5]. The Residual Cancer Burden (RCB) index is a quantitative non-dichotomous categorical measure of the residual tumour that considers the size of the residual tumour, the extent of nodal involvement, and the presence of metastatic disease [6]. The clinical and pathologic stage oestrogen receptor status and histologic grade (CPS-EG) score, based on clinical and pathological factors (i.e., clinical stage, oestrogen receptor status, and histologic grade [7]), is useful for stratifying patients and predicting treatment response, risk of recurrence, and the indication of PARP inhibitors in gBRCA1/2-mutated BC patients [7]. Currently, the most used method to assess pathological response to NAC is the binary classification of residual disease (RD) or complete response (pCR), defined as the absence of an invasive cancer component in the breast parenchyma (ypT0/is) and lymph nodes ypN0 [8]. The achievement of pCR after NAC in BC patients is increasingly recognised as a relevant prognostic parameter associated with long-term outcome, and is currently guiding escalation/de-escalation strategies in the post-NAC setting [9].

These findings, together with the revolution in BC treatment in recent decades, which has led to more personalised, less invasive, and more effective treatments [10], pave the way for a potential paradigm shift in patient management. Recent data suggest that, in well-selected cases with an optimal response, surgery can be omitted while still maintaining comparable outcomes [11]. This novel approach could improve quality of life, reduce treatment-related morbidity, and optimise cost-effectiveness [11]. In this context, the identification of BC patients with a higher probability of achieving pCR is of paramount importance.

[^18^F]Fluorodeoxyglucose positron emission tomography/computed tomography ([^18^F]FDG PET/CT) has a significant predictive value for disease recurrence and survival in BC patients [12,13]. However, the diagnostic performance of [^18^F]FDG PET in predicting pCR is still undetermined, particularly in relation to the RCB index and the CPS-EG score [13]. Although previous studies have proposed threshold values for semiquantitative PET parameters that can be used for identifying responders to NAC [14], these have not yet been widely adopted in clinical practice. This is primarily related to significant variations in study design, patient selection, the timing of PET scans, and the metabolic parameters evaluated (e.g., SUVmax, %ΔSUVmax, TLG or MTV) [14].

The objective of the present study is to investigate the role of baseline and preoperative [^18^F]FDG PET/CT as a non-invasive imaging modality to identify responders to NAC, according to the pCR/RD and RCB classifications, in a prospective cohort of BC patients.

## 2. Materials and Methods

### 2.1. Study Design and Participants

All newly diagnosed BC patients who were eligible for NAC followed by surgery according to standard clinical practice and who were referred to the Breast Unit were consecutively screened and invited to participate to this prospective observational cohort study. Eligible participants were female patients aged 18 years or older with histologically confirmed BC diagnosis, clinical TNM stage I–III, and who were considered suitable candidates for NAC followed by surgery according to standard clinical practice. Prior to enrolment, written informed consent was obtained from all participants. This sub-analysis included all recruited patients (period of recruitment between May 2019 and May 2022) who underwent baseline staging and preoperative restaging with whole-body [^18^F]FDG PET/CT.

All procedures were performed according to the Declaration of Helsinki. This study was approved by the Institutional Independent Ethics Committee (protocol identification number: ONC/OSS-02/2019). This cohort study followed the Strengthening the Reporting of Observational Studies in Epidemiology (STROBE) reporting guidelines [15].

### 2.2. NAC Regimens of Study Population

Sequential treatment with anthracycline- and taxane-based chemotherapy included, initially, regardless of BC subtype, four cycles of doxorubicin and cyclophosphamide (AC regimen). Subsequently, treatment was specific for each subtype: patients with Hormone Receptor positive/Human Epidermal Growth Factor Receptor 2 negative (HR+/HER2−) BC received docetaxel every three weeks for four cycles, patients with HR−/HER2+ BC received docetaxel plus trastuzumab for four cycles, and patients with triple-negative BC (TNBC) were treated with weekly carboplatin plus paclitaxel for 12 administrations.

### 2.3. Data Collection

We collected baseline clinicopathological data, including age, hormone receptor (HR) and Human Epidermal Growth Factor Receptor 2 (HER2) status, Ki-67 index, tumour grading, and clinical TNM stage according to the 8th edition [16]. Post-NAC data, including pathological TNM staging according to the 8th edition [16,17] and treatment response were also recorded. The final pathology served as the reference standard to assess response to NAC. Each patient was categorised as either pCR or RD [6], and the RCB index was also calculated. The categories of the RCB classification were defined as follows: RCB-0 as complete pathological response, RCB-I as minimal residual disease, RCB-II as moderate residual disease, and RCB-III as extensive residual disease [18].

### 2.4. [^18^F]FDG PET/CT Acquisition and Image Analysis

Whole-body PET/CT images were acquired 60 min after intravenous injection of 350–550 MBq [^18^F]FDG. PET/CT images were acquired using three different integrated scanners: Siemens Biograph 6 LSO (Siemens, Erlangen, Germany), General Electric Discovery 690 (General Electric Healthcare, Waukesha, WI, USA), and Siemens Vision (Siemens, Erlangen, Germany). All PET/CT scanners were accredited by the European Association of Nuclear Medicine (EANM, Vienna, Austria) Research Ltd. (EARL, Vienna, Austria) quality programme. Details about scanners and acquisition protocol are provided in Supplementary Table S1. Qualitative visual and semi-quantitative analyses were performed at both baseline and preoperative assessments on a dedicated PET/CT workstation (Advantage Workstation 4.6, GE Healthcare, Milwaukee, WI, USA) using the PET-VCAR (Volume Computer-Assisted Reading) software (GE Healthcare, Milwaukee, WI, USA). Both qualitative and semi-quantitative analyses were performed blinded to the results of the histopathological assessments. PET/CT scans were visually classified as positive if at least one lesion showed significant [^18^F]FDG uptake compared to physiological uptake in referent organs (i.e., mediastinum), uncertain if moderate uptake was observed, and negative if no significant [^18^F]FDG uptake was detected. The primary tumour and lymph nodes were separately visually assessed at patient-level both on baseline and preoperative PET/CT scans.

For semi-quantitative analysis, the primary tumour volume of interest (VOI) was semi-automatically segmented (threshold of 40% of the SUVmax). For multifocal disease, a single VOI was drawn to include all lesions. The maximum standardised uptake value (SUVmax) and the metabolically active tumour volume (MTV) were extracted from each VOI. Background SUVmean was extracted from the contralateral healthy breast parenchyma. The tumour-to-background ratio (TBR) was calculated by dividing the primary tumour SUVmax by the background SUVmean. Delta SUVmax (ΔSUVmax) was calculated as the difference in SUVmax between baseline and preoperative assessment.

### 2.5. Statistical Analysis

A summary of the general characteristics of the population was provided using frequency tables and ratios. Non-parametric continuous variables are reported as median with interquartile range (IQR). Categorical variables are expressed as frequencies and percentages. The Wilcoxon rank-sum test, the Kruskal–Wallis test, and the Bonferroni test for correction of multiple comparisons were employed to evaluate discrepancies in continuous non-parametric data between groups. Spearman’s rank correlation coefficient was employed to evaluate the relationships between continuous non-parametric variables. The Pearson chi-square test was used to compare differences in proportions of categorical variables. Diagnostic performance metrics of preoperative PET/CT (sensitivity, specificity, positive and negative predictive values, and accuracy) were calculated using histopathology as the reference standard, based on both pCR/RD and RCB index categories. For the RCB index, true residual disease was defined as RCB I, II, or III. The optimal cut-off value of preoperative SUVmax for predicting pCR/RD was determined using the Youden index. Univariate and multivariate logistic regression analyses were conducted to identify predictors of NAC response (pCR/RD and RCB-0/RCB-I/RCB-II/RCB-III), incorporating both clinical variables (i.e., age, BC subtypes, clinical TNM stage, and ki-67 index) and PET parameters at baseline and preoperative assessment (baseline MTV, baseline SUVmax, and preoperative SUVmax). The likelihood ratio chi-square test and the pseudo-R-squared value were employed to assess the model fit for both analyses. Furthermore, the odds ratios were calculated for each predictor, along with their 95% confidence intervals and *p*-values. The following classification performance metrics were calculated: sensitivity, specificity, positive predictive value, negative predictive value, and accuracy. All analyses involving RCB index as the outcome variable excluded patients for whom the RCB classification was unavailable. The level of statistical significance was set at *p* < 0.05. All statistical analyses were conducted using Stata MP software (version 17.0; StataCorp LP, College Station, TX, USA).

## 3. Results

### 3.1. Baseline Patients’ Characteristics

We analysed 133 patients with BC who met all of the inclusion criteria. Table 1 summarises the baseline clinicopathological characteristics.

### 3.2. Response to NAC Histopathological Assessment

Sixty-four patients achieved pCR following NAC. The RCB index was assessed in 129 out of 133 patients (RCB-0 = 59, RCB-I = 6, RCB-II = 52, and RCB-III = 12). In two cases, the RBC index was not determined due to the presence of lymph node disease alone; in the remaining two patients, surgery was performed at another institution which did not calculate the RCB. No significant differences in response to NAC were observed among different BC subtypes, regardless of the method to assess NAC response (*p* = 0.72 for pCR/RD and *p* = 0.436 for RCB), as shown in Supplementary Tables S2 and S3.

### 3.3. [^18^F]FDG PET/CT Qualitative Visual Analysis

Baseline and preoperative PET/CT results at patient-level of visual assessment are provided in Table 2.

No significant correlation was found between lymph node involvement at baseline PET/CT and response to NAC (Pearson chi2 = 2.17, *p* = 0.337).

Overall, 86/133 patients demonstrated a complete metabolic response to NAC at preoperative scan. The results of the visual analysis of preoperative PET/CT for NAC response by pCR/RD and RCB index are shown in Supplementary Table S4.

**Figure 1:**
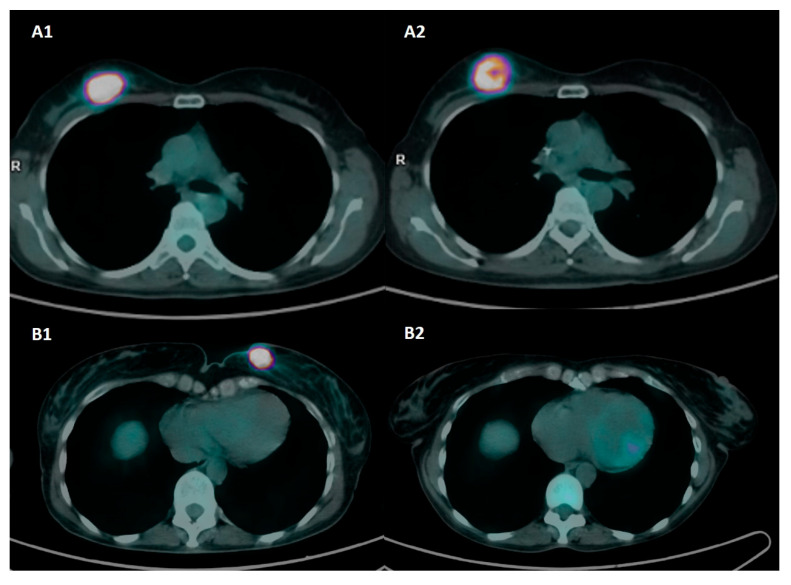
Axial [^18^F]FDG-PET/CT images at baseline and preoperative assessment in two patients with triple-negative BC and different response to NAC. Patient A was a 50-year-old patient with biopsy-confirmed invasive breast cancer, G3, ER: 0%, PgR: 0%, Ki67: 80%, HER2-positive, and axillary pathological lymph nodes (stage II). Baseline PET images (**A1**) show a pathological nodule with intense tracer uptake in the upper external quadrant of the right breast (SUVmax 46.8). Preoperative PET images (**A2**) show a partial metabolic response to NAC (SUVmax 31.1). Definitive histopathology after QUART + BLS was ypT2 N1a and classified as RD and RCB-III. Patient B was a 55-year-old patient with biopsy-proven invasive breast cancer, G2, ER: 0%, PgR: 0%, Ki67: 38%, HER2-, stage II. Baseline PET images (**B1**) show a pathological nodule with intense tracer uptake in the upper inner quadrant of the left breast (SUVmax 44.7). Preoperative PET images (**B2**) show a complete metabolic response to NAC (SUVmax 1.1). Definitive histopathology after QUART + BLS was ypT0 N0 and classified as pCR and RCB-0.

The sensitivity and specificity of preoperative PET/CT were 62.3% and 93.8%, respectively, based on the pCR/RD classification, whereas when the RCB index was considered, the sensitivity and specificity were 58.6% and 94.9%, respectively. The diagnostic performance of the qualitative analysis is presented in Supplementary Table S5. An example of a different response to NAC at preoperative PET/CT is provided in Figure 1.

### 3.4. [^18^F]FDG PET/CT Semi-Quantitative Analysis

**Figure 2:**
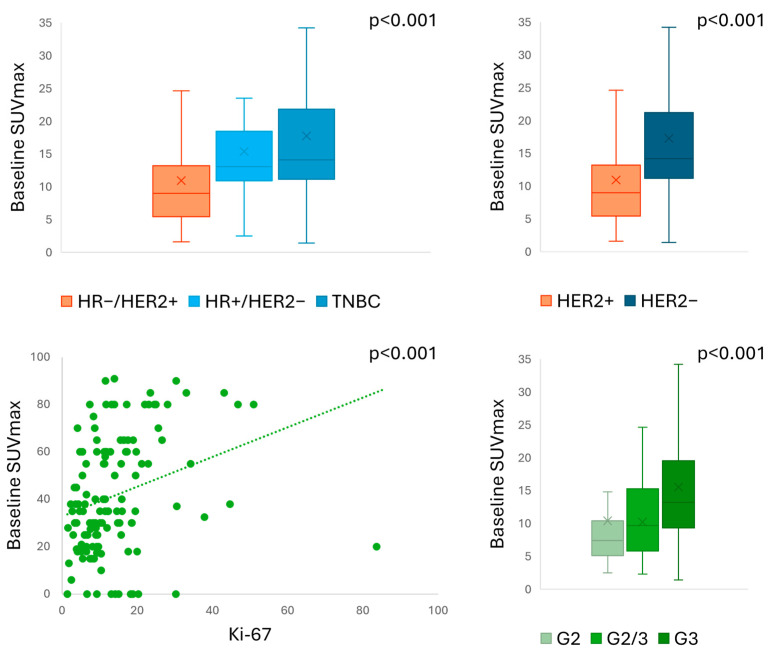
Baseline SUVmax according to pathological characteristics.

The PET semiquantitative parameters included in our study are summarised in Supplementary Table S6, showing significant differences between baseline and preoperative assessments (*p* < 0.001 for all). No significant difference in SUVmax was found between different PET scanners both at baseline and preoperative assessment (*p* = 0.48 and *p* = 0.78, respectively). Baseline SUVmax significantly correlated with tumour characteristics (Figure 2), including BC subtype (*p* < 0.001), HER2 expression (*p* < 0.001), tumour grading (*p* < 0.001), and Ki-67 index (*p* < 0.001).

A multiple-comparison analysis revealed significant differences in baseline SUVmax only between HR-/HER2+ and triple-negative BC (TNBC) subtypes (*p* = 0.003). Similar correlations were found for TBR with BC subtype (*p* = 0.005), HER2 expression (*p* = 0.002), tumour grading (*p* < 0.001), and Ki-67 index (*p* < 0.001). Supplementary Table S7 provides a summary of PET results according to different pathological characteristics. Baseline SUV parameters did not distinguish responders from non-responders to NAC by pCR (*p* = 0.39 for SUVmax, *p* = 0.59 for TBR, *p* = 0.13 for MTV) or by RCB index (*p* = 0.134 for SUVmax, *p* = 0.42 for TBR) (Supplementary Table S8). We found a significant difference in baseline MTV according to RCB index (*p* = 0.01), but in the multiple-comparison analysis, we were unable to distinguish between different RCB classes (*p* = 1.00 for all).

Preoperative SUVmax was correlated only with baseline Ki-67 index (*p* = 0.019) and clinical TNM stage (*p* = 0.035). Preoperative TBR was not correlated with any variable. There was no significant difference in SUVmax of the primary tumour based on lymph node involvement at either baseline or preoperative assessment.

**Figure 3:**
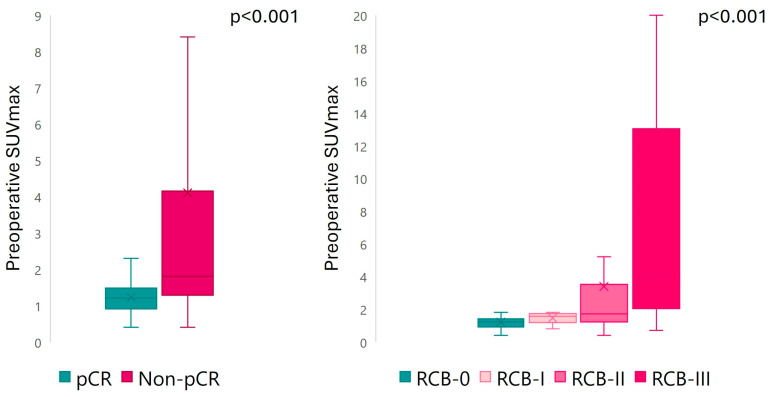
Preoperative SUVmax according to response to NAC.

Preoperative SUVmax and preoperative TBR were significantly higher in non-responders compared to responders, regardless of the classification method for NAC assessment (*p* < 0.001 for all) (Figure 3). A multiple-comparison analysis found significant differences in preoperative SUVmax between RCB-III and RCB-0 (*p* < 0.001), RCB-I (*p* = 0.005), and RCB-II (*p* = 0.001), as well as between RCB-0 and RCB-II (*p* = 0.022). Similarly, preoperative TBR significantly differed between RCB-III and RCB-0 (*p* < 0.001), RCB-I (*p* = 0.017), and RCB-II (*p* = 0.005), and between RCB-0 and RCB-II (*p* = 0.022). No significant differences in preoperative MTV were found according to pCR/RD (*p* = 0.5) and RCB index (*p* = 0.4) (Supplementary Table S9). The ΔSUVmax only correlated with pCR/RD (*p* = 0.008), but was unable to discriminate RCB categories (*p* = 0.06) (Supplementary Table S10). The preoperative SUVmax cut-off value, empirically set at 31 to discriminate between responders and non-responders, presented an area under the curve of 0.50 (0% sensitivity, 100% specificity), indicating no discriminatory power in this cut-off.

A subgroup analysis showed that preoperative SUVmax was significantly higher in patients with RD compared to those with pCR across different BC subtypes (*p* = 0.011 for HR+/HER2-, *p* = 0.0015 for HR-/HER2+, and *p* = 0.0004 for TNBC). Patients with RCB-III exhibited higher preoperative SUVmax than other classes (*p* = 0.04 for HR+/HER2-, *p* = 0.0015 for HR-/HER2+, and *p* = 0.0011 for TNBC). By subclassifying patients based on HER2 expression, preoperative SUVmax was significantly higher in patients with RD compared to those with pCR in both HER2 classes (Supplementary Figure S1). Similarly, patients with RCB-III also showed higher preoperative SUVmax than other classes (*p* < 0.001 for all). Complete preoperative SUVmax results according to NAC response, BC subtypes, and HER2 expression are presented in Supplementary Tables S11 and S12.

Preoperative SUVmax emerged as the only significant predictor of response to NAC for both pCR and RCB index (*p* < 0.001). None of the other clinical, pathological, or PET variables were statistically significant predictors. The detailed results for each predictor are summarised in Table 3. The model for pCR prediction demonstrated a significant overall association between the predictors and the likelihood of achieving pCR (chi2 = 48.68, *p* < 0.001), with a pseudo-R2 value of 0.27. Similarly, the model for RCB index prediction showed a significant overall association between the predictors and the likelihood of RCB classification (chi2 = 45.88, *p* < 0.0001), with a pseudo-R2 value of 0.27. The overall accuracy of the models for pCR and RCB classification were 71.32% and 72.80%, respectively. The classification performance of the models is detailed in Supplementary Table S13.

## 4. Discussion

Our study, demonstrating the effectiveness of preoperative [^18^F]FDG PET/CT in assessing the efficacy of NAC, highlights the significant value of molecular imaging in guiding BC patient management. Firstly, the visual assessment of preoperative imaging proved to have high specificity in identifying patients with residual disease after NAC. Furthermore, responders and non-responders exhibited significantly different preoperative SUVmax and TBR, regardless of the treatment response classification (i.e., pCR/RD or RCB index) across different BC subtypes and HER2 expression patterns. However, unlike previous reports [14], in our cohort, the SUVmax cut-off of 31 yielded an AUC of only 0.50. This highlights the lack of clinical utility of SUVmax as a stand-alone discriminator of treatment response, reflecting both BC heterogeneity and SUVmax variability. Indeed, the inherent limitations of SUVmax make identifying a clinical meaningful cut-off challenging [18], especially outside specific harmonisation programmes [12]. Additionally, tumour subtype and type of treatment should be considered in BC patients undergoing NAC, as TNBC presents higher [^18^F]FDG uptake than others molecular subtypes [19,20], a finding confirmed by our results. Moreover, SUV values showed higher reduction with lapatinib + trastuzumab than with trastuzumab alone [21]. All of these factors may contribute to explaining the difference between our results and previous data in identifying a cut-off in [^18^F]FDG uptake. Advanced quantitative methods, such as radiomics, have been proposed to overcome the limitations of conventional PET parameters, as they can capture intra-tumoural heterogeneity, which may reflect the underlying tumour biology. However, in a previous study, we explored the use of radiomics in this clinical setting and found no significant improvement in performance (mean accuracy = 0.50 ± 0.11) [22].

In our prospective cohort, preoperative PET was performed using a standardise timing after NAC completion and visual assessment was used to rate an exam as negative or positive. However, the optimal timing and the appropriate PET parameter for assessing NAC response remain uncertain [12]. A recent meta-analysis found no significant difference between interim and preoperative PET assessments, irrespective of the timing of the interim assessment [14]. Moreover, some randomised clinical trials have explored interim PET/CT for de-escalation treatment strategies in HER2+ BC [23,24]. Recently, the PHERGain trial proposed a PET-adapted strategy to guide treatment decisions, allowing for chemotherapy omission for patients responsive to exclusive HER2-targeted therapies. This PET-guided de-escalation approach showed comparable three-year disease-free survival rates and a more favourable toxicity profile [25], identifying a Delta SUVmax cut-off of ≥77% as the optimal threshold for pCR classification [24]. These findings emphasise the potential of PET to predict long-term outcomes and to guide the tailoring of therapeutic strategies based on individual responses.

Baseline PET parameters, confirming our previous findings [22], were not correlated with NAC response in our population. This was in contrast with previous report by Kazerouni et al., who identified a significant association between baseline SUVmax and RCB-classes in a small cohort of BC patients, mainly luminal B and TNBC, treated with NAC [26]. Similarly, Seban et al. identified the baseline PET parameter as a significant predictor of response to NAC or chemo-immunotherapy [27] in a population of TNBC. However, baseline SUVmax exhibited significant correlations with a range of tumour characteristics, including BC subtype, HER2 expression, tumour grading, and Ki-67 index. These correlations suggest that SUVmax is indicative of the underlying tumour biology, in accordance with previous studies that have established a correlation between high SUVmax and aggressive tumour characteristics [20].

We found preoperative SUVmax to be the only predictor of NAC response in multivariate logistic regression analyses. The predictive value of PET/CT varied significantly depending on the specific BC subtype and HER2 expression pattern under consideration. These findings suggest that it may be of particular benefit in identifying non-responders within specific BC subtypes. For instance, patients with HER2− subtypes and RD showed a substantially higher preoperative SUVmax in comparison to those who exhibited a pCR (2.5 vs. 1). Considering these findings, several ongoing clinical trials (NCT04595565, NCT05633654, NCT05629585, NCT04622319, NCT04873362) are evaluating escalation therapies for patients with residual disease after NAC. Furthermore, our study demonstrates a significant variation in preoperative SUVmax between the different RCB classes, with a notable divergence between RCB-III and RCB-II compared to the other lower groups. Our results agree with Kazerouni et al., who used multiparametric PET imaging at baseline, interim, and preoperative time points and showed that patients with RCB-I/II had a greater reduction in SUVmax and other PET parameters compared to RCB-II/III [26]. Although Delta SUVmax has been identified as a valuable biomarker for response to NAC [14], it was not useful for RCB class discrimination in our cohort.

It should be noted that the present study is not without limitations. Firstly, we did not identify specific cut-off values to accurately differentiate between response classes. The absence of a definitive cut-off value represents a significant challenge to the clinical application of PET/CT as a binary predictive tool. However, as also highlighted by current guidelines, no specific cut-off values for these metrics can be recommended due to lack of compliance with harmonising standards [12]. On the other hand, a notable strength of our study was the prospective design, standardised treatment schemes, and homogeneous timing for treatment assessment prior to surgery. This rigorous approach enhanced the reliability and generalisability of our findings.

## 5. Conclusions

In conclusion, preoperative [^18^F]FDG PET/CT demonstrated considerable potential for evaluating the efficacy of NAC in BC patients across different subtypes and HER2 expression patterns, regardless of the treatment response classification. Visual analysis showed high specificity, and semi-quantitative parameters provided additional valuable information. PET/CT imaging can support surgical decision-making by helping us to identify patients who have achieved pCR and those with extensive RD. Although PET/CT alone is insufficient to determine the omission of surgery, it can provide complementary information to inform individualised treatment strategies and potentially reduce the need for unnecessary surgical interventions.

## Figures and Tables

**Table 1:** Baseline patients’ characteristics.

Variables (*n* = 133)		N (%)	Median (IQR)
Age (y)			49.5 (42–57)
Molecular subtypes (n)	HR+/HER2−	13 (10)	
	HR−/HER2+	74 (55)	
	TNBC	46 (35)	
HER2 status (n)	Positive	74 (56)	
	Negative	59 (44)	
HR status (n)	Positive	13 (10)	
	Negative	120 (90)	
Ki67 (%)			35 (25–60)
Tumour grading (n)	G2	31 (23)	
	G2/3	19 (14)	
	G3	75 (57)	
	NA	8 (6)	
Clinical TNM stage (n)	I	5 (3)	
	II	104 (78)	
	III	22 (17)	
	NA	2 (2)	
Clinical lymph node status (n)	Negative	63 (47)	
	Positive	70 (53)	

**Table 2:** Baseline and preoperative results at a patient-level of visual assessment.

Site	Primary Tumour		Lymph Nodes		
[^18^F]FDG PET/CT	Positive	Negative	Positive	Negative	Uncertain
Baseline	130	3 *	76	25	32
Preoperative	43	90	12	117	4

* No [^18^F]FDG uptake in primary tumour; lymph node spread only.

**Table 3:** Univariate and multivariate logistic regression analysis for pCR/RD and RCB index prediction.

Variables	pCR/RD				RCB Index			
	Univariate		Multivariate		Univariate		Multivariate	
	OR	*p*-Value	OR	*p*-Value	OR	*p*-Value	OR	*p*-Value
Age	1 (0.97–1.03)	0.98	0.99 (0.94–1.02)	0.31	1 (0.97–1.04)	0.61	1 (0.99–1.08)	0.11
BC subtypes		0.72				0.77		
HR+/HER2−	Ref.		Ref.		Ref.		Ref.	
HR−/HER2+	1.4 (0.43–4.8)		1.6 (0.4–7.8)	0.46	0.78 (0.23–2.6)		0.7 (0.15–3.15)	0.63
TNBC	1.67 (0.4–5.87)		2.24 (0.46–11)	0.32	0.65 (0.18–2.3)		0.58 (0.1–3)	0.51
Ki-67	1 (0.98–1.01)	0.97	1 (0.98–1.03)	0.4	1.00 (0.98–1.01)	0.98	1 (0.97–1.01)	0.98
Stage		0.35				0.40		
I	Ref.		Ref.		Ref.		Ref.	0.40
II	0.66 (0.1–4.15)		1.35 (0.16–10.9)	0.78	1.7 (0.27–10.5)		0.99 (0.12–8.2)	
III	0.35 (0.05–2.5)		0.7 (0.67–7.67)	0.78	3 (0.40–22)		1.75 (0.14–21)	
Baseline MTV	0.99 (0.97–1.02)	0.73	1 (0.99–1.03)	0.29	1.00 (0.97–1.03)	0.88	0.97 (0.92–1.01)	0.19
Baseline SUVmax	1 (0.98–1.05)	0.33	1.04 (0.99–1.10)	0.1	0.99 (0.96–1.02)	0.52	0.97 (0.93–1.01)	0.18
Preoperative SUVmax	0.29 (0.15–0.56)	<0.001 *	0.26 (0.13–0.5)	<0.001 *	3.74 (1.8–7.7)	<0.001 *	4.2 (1.99–8.9)	<0.001 *

* statistically significant values.

## Data Availability

Data associated with this article are stored in the Zenodo repository (zenodo.org) and available upon request due to privacy issues. DOI: 10.5281/zenodo.15729382.

## Supplementary Materials

The following supporting information can be downloaded at: https://www.mdpi.com/article/10.3390/cancers17132133/s1, Figure S1: Preoperative SUVmax according to response to NAC in different HER2 expression classes; Table S1: Scanner characteristics and acquisition protocols; Table S2: Response to NAC assessment according to pCR and RCB criteria; Table S3: Response to NAC according to BC subtypes; Table S4: Visual analysis of preoperative PET/CT and response to NAC; Table S5: Diagnostic performance metrics of visual analysis in identifying residual disease at preoperative PET/CT; Table S6: SUV parameters in the entire cohort (n = 133); Table S7: SUV values according to pathological characteristics; Table S8: Baseline PET parameters according to response to NAC; Table S9: Preoperative PET parameters according to response to NAC; Table S10: ΔSUVmax values according to response to NAC; Table S11: Preoperative SUVmax of the primary tumour according to BC subtypes; Table S12: Preoperative SUVmax of the primary tumour according to HER2 expression; Table S13: Classification performance of multivariate logistic regression analysis for pCR and RCB index.
