# Supplementary material for: Using [18F]FDG PET/CT to Identify Optimal Responders to Neoadjuvant Therapy in Breast Cancer—Results from a Prospective Patient Cohort
Source: Cancers (Basel). 2025 Jun 25;17(13):2133. doi: 10.3390/cancers17132133 (PMC12248987; doi:10.3390/cancers17132133)
Supplement: Supplementary file 1 [file cancers-17-02133-s001.zip › Supplementary Table S1.pdf]

Table S1: Scanner characteristics and acquisition protocols.

Acquisition parameters	Biograph – Siemens		Discovery 690 – General Electric		Vision - Siemens	
	PET	CT	PET	CT	PET	CT
**[^{18}F]FDG activity (MBq)***	350–550	–	350–550	–	350–550	–
**Min/bed position**	2.5	–	2	–	Flow-motion	–
**Crystal**	LSO	–	LYSO	–	LSO	–
**Reconstruction**	Iterative	–	Iterative, TOF Sharp IR	–	Iterative, Ultra HD	Iterative
**Matrix (pixels)**	128×128	512×512	256×256	512×512	440	512
**Resolution (mm)**	5.3×5.3	0.98×0.98	2.73×2.73	1.37×1.37	1.65x1.65	0.6x0.6
**Slice thickness (mm)**	2.0	4.0	3.27	3.27	2.0	–
**Slices**	–	6	–	64	–	64
**Voltage (kV)**	–	130	–	140	–	120
**Tube current (mA)**	–	95	–	140	–	–
**Reconstruction**		–	Iterative, TOF Sharp IR	–	Iterative, TOF Ultra HD	–
*Administered activity was calculated according to the European Association of Nuclear Medicine (EANM) guidelines 2.0 (Boellaard et al., 2014)						
