# Supplementary material for: Using [18F]FDG PET/CT to Identify Optimal Responders to Neoadjuvant Therapy in Breast Cancer—Results from a Prospective Patient Cohort
Source: Cancers (Basel). 2025 Jun 25;17(13):2133. doi: 10.3390/cancers17132133 (PMC12248987; doi:10.3390/cancers17132133)
Supplement: Supplementary file 1 [file cancers-17-02133-s001.zip › Supplementary Table S10.pdf]

Table S10: ΔSUVmax values according to response to NAC.

**Variables**		**ΔSUVmax**	**p-value**
**pCR**	pCR	11 (5 – 18)	0.008*
	RD	8 (4 – 13)	
**RCB index**	RCB-0	11 (5 – 17.5)	0.06
	RCB-I	14.5 (5-21.5)	
	RCB-II	7 (4-12)	
	RCB-III	8.5 (6-15)	
