# Supplementary material for: Using [18F]FDG PET/CT to Identify Optimal Responders to Neoadjuvant Therapy in Breast Cancer—Results from a Prospective Patient Cohort
Source: Cancers (Basel). 2025 Jun 25;17(13):2133. doi: 10.3390/cancers17132133 (PMC12248987; doi:10.3390/cancers17132133)
Supplement: Supplementary file 1 [file cancers-17-02133-s001.zip › Supplementary Table S11.pdf]

Table S11: Preoperative SUVmax of the primary tumour according to BC subtypes.

		**HR+/HER2-**	**p-value**	**HR-/HER2+**	**p-value**	**TNBC**	**p-value**
**pCR/RD**	pCR	1.2 (0.7 – 1.2)	0.011*	1.2 (0.9 – 1.4)	0.0015*	1.1 (0.95 – 1.55)	0.0004*
	RD	2.15 (1.6 -2.3)		1.7 (1.2 – 3.1)		4 (1.4 – 10.2)	
**RCB index**	RCB-0	1.2 (0.7 – 1.2)	0.04*	1.2 (0.9 – 1.4)	0.0015*	1.15 (0.9 – 1.6)	0.0011*
	RCB-I	NA		1.55 (1.3 – 1.7)		NA	
	RCB-II	2.3 (1.4 – 2.3)		1.6 (1.2 – 2.4)		1.7 (1.1 – 8.2)	
	RCB-III	1.9 (1.8 – 2)		3.5 (2.1 – 5.8)		14.35 (6.35 – 25.55)	
