# Supplementary material for: Using [18F]FDG PET/CT to Identify Optimal Responders to Neoadjuvant Therapy in Breast Cancer—Results from a Prospective Patient Cohort
Source: Cancers (Basel). 2025 Jun 25;17(13):2133. doi: 10.3390/cancers17132133 (PMC12248987; doi:10.3390/cancers17132133)
Supplement: Supplementary file 1 [file cancers-17-02133-s001.zip › Supplementary Table S12.pdf]

Table S12: Preoperative SUVmax of the primary tumour according to HER2 expression.

		**HER2-**	**p-value**	**HER2+**	**p-value**
**pCR/RD**	pCR	1 (1– 1.5)	0.0001*	1 (1 – 1.5)	0.0015*
	RD	2.5 (1.5 – 8.5)		2 (1– 3)	
**RCB index**	RCB-0	1 (1– 1.5)	0.0001*	1 (1 – 1.45)	0.0015*
	RCB-I	NA		1.5 (1.5 – 2)	
	RCB-II	2 (1.35 – 8)		2 (1 – 2.5)	
	RCB-III	6.5 (2 – 20)		3.5 (2 – 6)	
