# Supplementary material for: Using [18F]FDG PET/CT to Identify Optimal Responders to Neoadjuvant Therapy in Breast Cancer—Results from a Prospective Patient Cohort
Source: Cancers (Basel). 2025 Jun 25;17(13):2133. doi: 10.3390/cancers17132133 (PMC12248987; doi:10.3390/cancers17132133)
Supplement: Supplementary file 1 [file cancers-17-02133-s001.zip › Supplementary Table S2.pdf]

Table S2: Response to NAC assessment according to pCR and RCB criteria.

	**RCB-0**	**RCB-I**	**RCB-II**	**RCB-III**	**NA**
**pCR**	59	2	1	0	2
**RD**	0	4	51	12	2
