# Supplementary material for: Using [18F]FDG PET/CT to Identify Optimal Responders to Neoadjuvant Therapy in Breast Cancer—Results from a Prospective Patient Cohort
Source: Cancers (Basel). 2025 Jun 25;17(13):2133. doi: 10.3390/cancers17132133 (PMC12248987; doi:10.3390/cancers17132133)
Supplement: Supplementary file 1 [file cancers-17-02133-s001.zip › Supplementary Table S3.pdf]

Table S3: Response to NAC according to BC subtypes.

**Response to NAC**		**Molecular subtypes**				**Pearson chi2**	**p-value**
		HR+/HER2-	HR-/HER2+	TNBC	Tot		
**pCR/RD (n)**	pCR	5	35	23	63	0.66	0.72
	RD	8	39	23	70		
**RCB index (n)**	RCB-0	5	32	22	59	5.88	0.436
	RCB-I	0	6	0	6		
	RCB-II	6	28	18	52		
	RCB-III	2	6	4	12		
	NA	0	2	2	4		
