# Supplementary material for: Using [18F]FDG PET/CT to Identify Optimal Responders to Neoadjuvant Therapy in Breast Cancer—Results from a Prospective Patient Cohort
Source: Cancers (Basel). 2025 Jun 25;17(13):2133. doi: 10.3390/cancers17132133 (PMC12248987; doi:10.3390/cancers17132133)
Supplement: Supplementary file 1 [file cancers-17-02133-s001.zip › Supplementary Table S4.pdf]

Table S4: visual analysis of preoperative PET/CT and response to NAC.

	**Preoperative PET/CT results**	
**Reference standard**	**Negative (n)**	**Positive (n)**
**PCR (n)**	60	4
**RD (n)**	26	43
**RCB-0 (n)**	56	3
**RCB-I/II/III (n)**	29	41
