# Supplementary material for: Using [18F]FDG PET/CT to Identify Optimal Responders to Neoadjuvant Therapy in Breast Cancer—Results from a Prospective Patient Cohort
Source: Cancers (Basel). 2025 Jun 25;17(13):2133. doi: 10.3390/cancers17132133 (PMC12248987; doi:10.3390/cancers17132133)
Supplement: Supplementary file 1 [file cancers-17-02133-s001.zip › Supplementary Table S5.pdf]

Table S5: Diagnostic performance metrics of visual analysis in identifying residual disease at preoperative PET/CT.

	**Sensitivity (%)**	**Specificity (%)**	**PPV (%)**	**NPV (%)**	**Accuracy (%)**
**pCR/RD**	62.3 (49.8 – 73.3)	93.8 (84.8 – 98.3)	91.5 (79.6 – 97.6)	69.8 (58.9 – 79.2)	77.4 (69.4 -84.2)
**RCB index**	58.6 (46.2 – 70.2)	94.9 (85.9 – 98.9)	93.2 (81.3 – 98.6)	65.9 (54.8 – 75.8)	75.2 (66.8 - 82.4)
