# Supplementary material for: Using [18F]FDG PET/CT to Identify Optimal Responders to Neoadjuvant Therapy in Breast Cancer—Results from a Prospective Patient Cohort
Source: Cancers (Basel). 2025 Jun 25;17(13):2133. doi: 10.3390/cancers17132133 (PMC12248987; doi:10.3390/cancers17132133)
Supplement: Supplementary file 1 [file cancers-17-02133-s001.zip › Supplementary Table S6.pdf]

Table S6: SUV parameters in the entire cohort (n=133).

	Baseline	Preoperative	p-value
**SUVmax**	11.4 (6.5 - 17.3)	1.4 (1 - 2.1)	<0.001*
**TBR**	7.59 (4.39 - 14)	1.25 (1 - 2)	<0.001*
**MTV**	6.35 (3.8 - 11.7)	2.3 (1.9 - 2.8)	<0.001*
