# Supplementary material for: Using [18F]FDG PET/CT to Identify Optimal Responders to Neoadjuvant Therapy in Breast Cancer—Results from a Prospective Patient Cohort
Source: Cancers (Basel). 2025 Jun 25;17(13):2133. doi: 10.3390/cancers17132133 (PMC12248987; doi:10.3390/cancers17132133)
Supplement: Supplementary file 1 [file cancers-17-02133-s001.zip › Supplementary Table S7.pdf]

Table S7: SUV values according to pathological characteristics.

Variables		Baseline SUVmax	p-value	Baseline TBR	p-value	Preoperative SUVmax	p-value	Preoperative TBR	p-value
**BC subtype**	HR+/HER2-	11.5 (11- 19)	<0.001*	9.5 (5-5 - 14)	0.005*	1.5 (1–2.5)	0.453	1.4 (0.9-1.8)	0.357
	HR-/HER2+	9 (5.5 – 13.5)		6 (4 - 11)		1.4 (0.9-1.8)		1.25 (1-1.6)	
	TNBC	14 (11 - 22)		10 (6.5 - 22)		1.5 (1-4)		1.4 (1-4)	
**HER2 expression**	Positive	9 (5.5 -13.5)	<0.001*	5.7 (3.6-10.8)	0.002*	1.5 (1.1-2.5)	0.209	1.25 (1-1.6)	0.306
	Negative	14 (11-21.6)		10 (6 -18.5)		1.4 (0.9-1.8)		1.4 (1-3)	
**Ki-67**		rho 0.36	<0.001*	rho 0.325	<0.001*	rho 0.2	0.019*	rho 0.07	0.4
**Stage**	I	5.4 (5 - 13)	0.27	6 (5 - 19)	0.3	0.9 (0.8-0.9)	0.035*	1.3 (0.7-1.5)	0.49
	II	12 (7 - 18)		9 (4 - 15)		1.5 (1-1.8)		1.2 (1-2)	
	III	10 (6 - 16)		6 (4 - 11)		1.7 (1.1-2.3)		1.4 (1.1-1.9)	
**N status**	Negative	11 (6 - 16)	0.38	7 (4-18)	0.6	1.4 (0.9-2)	0.357	1.25 (1-2.6)	0.4
	Positive	11.5 (7 - 18)		9 (5-13)		1.5 (1.1-2.1)		1.25 (1.06-1.9)	
**Grading**	G2	7 (5 - 10)	<0.001*	5 (3 - 6.5)	<0.001*	1.4 (1.2-2.3)	0.93	1.3 (1.1-1.5)	0.76
	G2/3	11.5 (6 – 16)		7 (3 - 11)		1.3 (1-2.3)		1.25 (1-1.9)	
	G3	14 (9 - 20)		10 (5.5 - 18.5)		1.5 (1-1.95)		1.3 (1-2.8)	
