# Supplementary material for: Using [18F]FDG PET/CT to Identify Optimal Responders to Neoadjuvant Therapy in Breast Cancer—Results from a Prospective Patient Cohort
Source: Cancers (Basel). 2025 Jun 25;17(13):2133. doi: 10.3390/cancers17132133 (PMC12248987; doi:10.3390/cancers17132133)
Supplement: Supplementary file 1 [file cancers-17-02133-s001.zip › Supplementary Table S8.pdf]

Table S8: baseline PET parameters according to response to NAC.

Variables		Baseline SUVmax	p-value	Baseline TBR	p-value	Baseline MTV	p-value
**pCR**	pCR	12 (6 - 19)	0.39	9 (5 – 14)	0.59	6 (3 – 11)	0.13
	RD	10 (7 - 16)		7 (4.35 – 15)		8 (4 – 12.5)	
**RCB index**	RCB-0	12 (6 – 19)	0.134	9 (4 – 14)	0.42	6 (3 – 11.5)	0.01*
	RCB-I	16 (7 - 23)		9.5 (4 – 23)		3.5 (1.6 – 5)	
	RCB-II	9 (6.5 - 15)		7 (4 – 13)		7 (4 – 11.6)	
	RCB-III	15 (10 - 25)		10 (6 – 18)		10 (7 – 14)	
