# Supplementary material for: Using [18F]FDG PET/CT to Identify Optimal Responders to Neoadjuvant Therapy in Breast Cancer—Results from a Prospective Patient Cohort
Source: Cancers (Basel). 2025 Jun 25;17(13):2133. doi: 10.3390/cancers17132133 (PMC12248987; doi:10.3390/cancers17132133)
Supplement: Supplementary file 1 [file cancers-17-02133-s001.zip › Supplementary Table S9.pdf]

Table S9: preoperative PET parameters according to response to NAC.

Variables		Preoperative SUVmax	p-value	Preoperative TBR	p-value	Preoperative MTV	p-value
**pCR**	pCR	1.2 (0.9 – 1.4)	<0.001*	1.09 (0.97 - 1.3)	<0.001*	2.3 (2 – 3)	0.5
	RD	1.8 (1.3 – 4.0)		1.7 (1.2 - 4.3)		2.3 (2 – 3)	
**RCB index**	RCB-0	1.2 (0.9 – 1.4)	<0.001*	1.09 (0.93 - 1.3)	<0.001*	2.5 (2 – 3)	0.4
	RCB-I	1.5 (1.3 – 1.7)		1.25 (1.08 - 1.6)		2.5 (2 – 3)	
	RCB-II	1.7 (1.2 – 3.2)		1.5 (1.07 - 3.45)		2.3 (2 – 3)	
	RCB-III	3.9 (2 -12)		3 (1.7 - 12)		3 (2 – 5)	
