# Supplementary figures and images for: Using [18F]FDG PET/CT to Identify Optimal Responders to Neoadjuvant Therapy in Breast Cancer—Results from a Prospective Patient Cohort
Source: Cancers (Basel). 2025 Jun 25;17(13):2133. doi: 10.3390/cancers17132133 (PMC12248987; doi:10.3390/cancers17132133)

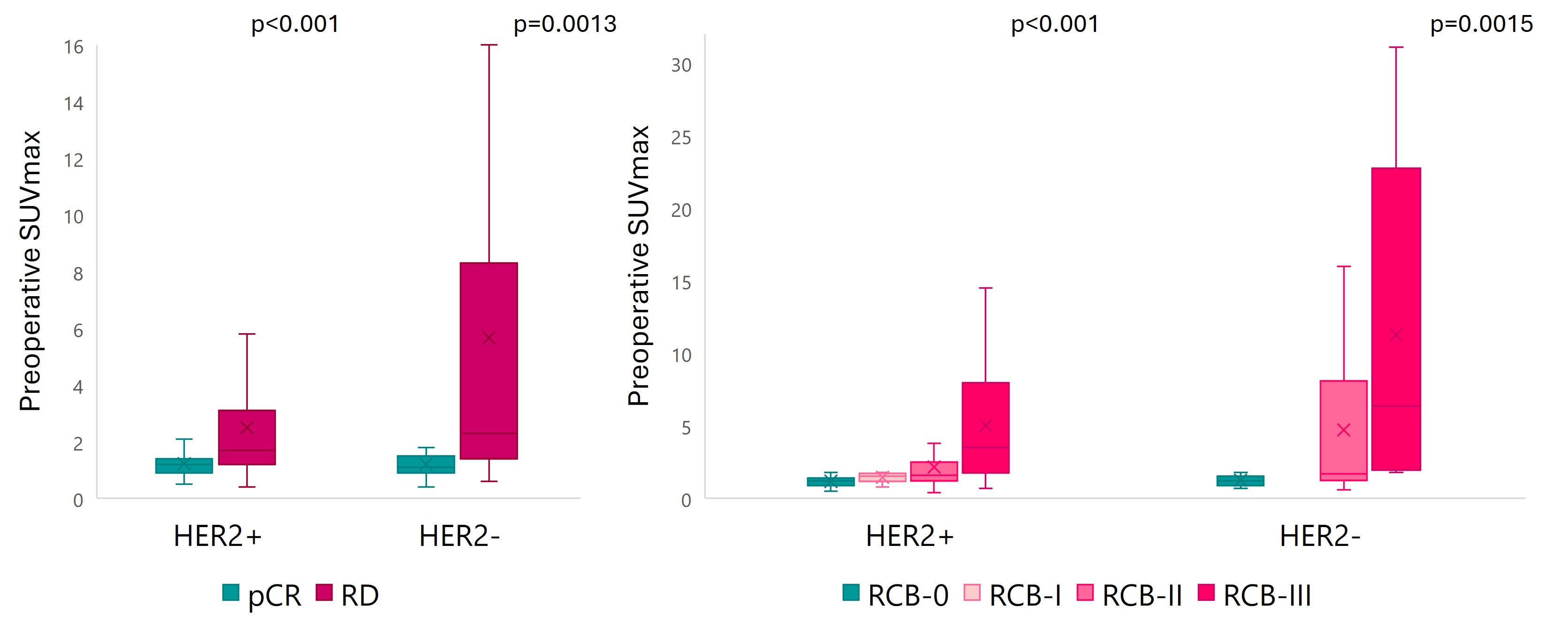

Supplement: Supplementary file 1 [file cancers-17-02133-s001.zip › Supplementary Figure S1.jpg]
